# Patient Partnership Tools to Support Medication Safety in Community-Dwelling Older Adults: Protocol for a Nonrandomized Stepped Wedge Clinical Trial

**DOI:** 10.2196/57878

**Published:** 2024-04-29

**Authors:** Yan Xiao, Kimberley G Fulda, Richard A Young, Z Noah Hendrix, Kathryn M Daniel, Kay Yut Chen, Yuan Zhou, Jennifer L Roye, Ludmila Kosmari, Joshua Wilson, Anna M Espinoza, Kathleen M Sutcliffe, Samantha I Pitts, Alicia I Arbaje, Michelle A Chui, Somer Blair, Dawn Sloan, Masheika Jackson, Ayse P Gurses

**Affiliations:** 1 College of Nursing and Health Innovation University of Texas at Arlington Arlington, TX United States; 2 College of Engineering University of Texas at Arlington Arlington, TX United States; 3 Department of Family Medicine and Osteopathic Manipulative Medicine and North Texas Primary Care Practice-Based Research Network (NorTex) University of North Texas Health Science Center Fort Worth, TX United States; 4 Family Medicine Residency Program John Peter Smith Health Network Fort Worth, TX United States; 5 College of Business University of Texas at Arlington Arlington, TX United States; 6 College of Liberal Arts University of Texas at Arlington Arlington, TX United States; 7 Carey Business School Johns Hopkins University Baltimore, MD United States; 8 School of Medicine Johns Hopkins University Baltimore, MD United States; 9 Bloomberg School of Public Health Johns Hopkins University Baltimore, MD United States; 10 School of Nursing Johns Hopkins University Baltimore, MD United States; 11 School of Pharmacy University of Wisconsin-Madison Madison, WI United States; 12 Office of Clinical Research John Peter Smith Health Network Fort Worth, TX United States; 13 Whiting School of Engineering Johns Hopkins University Baltimore, MD United States

**Keywords:** primary care, medication safety, communication, patient engagement, human factors, medication, safety, engagement, support, community dwelling, older adults, elderly, protocol, patient safety, self-management, ambulatory, medications, tool, tools, effective, medication safety, data collection, decision, decision making, care, self-efficacy, engagement tool

## Abstract

**Background:**

Preventable harms from medications are significant threats to patient safety in community settings, especially among ambulatory older adults on multiple prescription medications. Patients may partner with primary care professionals by taking on active roles in decisions, learning the basics of medication self-management, and working with community resources.

**Objective:**

This study aims to assess the impact of a set of patient partnership tools that redesign primary care encounters to encourage and empower patients to make more effective use of those encounters to improve medication safety.

**Methods:**

The study is a nonrandomized, cross-sectional stepped wedge cluster-controlled trial with 1 private family medicine clinic and 2 public safety-net primary care clinics each composing their own cluster. There are 2 intervention sequences with 1 cluster per sequence and 1 control sequence with 1 cluster. Cross-sectional surveys will be taken immediately at the conclusion of visits to the clinics during 6 time periods of 6 weeks each, with a transition period of no data collection during intervention implementation. The number of visits to be surveyed will vary by period and cluster. We plan to recruit patients and professionals for surveys during 405 visits. In the experimental periods, visits will be conducted with two partnership tools and associated clinic process changes: (1) a 1-page visit preparation guide given to relevant patients by clinic staff before seeing the provider, with the intention to improve communication and shared decision-making, and (2) a library of short educational videos that clinic staff encourage patients to watch on medication safety. In the control periods, visits will be conducted with usual care. The primary outcome will be patients’ self-efficacy in medication use. The secondary outcomes are medication-related issues such as duplicate therapies identified by primary care providers and assessment of collaborative work during visits.

**Results:**

The study was funded in September 2019. Data collection started in April 2023 and ended in December 2023. Data was collected for 405 primary care encounters during that period. As of February 15, 2024, initial descriptive statistics were calculated. Full data analysis is expected to be completed and published in the summer of 2024.

**Conclusions:**

This study will assess the impact of patient partnership tools and associated process changes in primary care on medication use self-efficacy and medication-related issues. The study is powered to identify types of patients who may benefit most from patient engagement tools in primary care visits.

**Trial Registration:**

ClinicalTrials.gov NCT05880368; https://clinicaltrials.gov/study/NCT05880368

**International Registered Report Identifier (IRRID):**

DERR1-10.2196/57878

## Introduction

### Background

Health care encounters are opportunities for health care professionals (HCPs) to work productively with patients and families to address risks in medication use in ambulatory settings [1,2]. This study assesses a systematic redesign of time-limited primary care encounters to improve medication safety. Adverse drug events (ADEs) account for 6.1 emergency department visits per 1000 population each year in the United States and 38.6% of these visits require hospitalization [3]. Medication safety is recognized as a significant area for improvement by the World Health Organization (“Medication without Harm”) [4]. The National Action Plan for ADE Prevention [5] highlighted ADEs associated with insulins, opioids, and anticoagulants, such as those from hypoglycemia, opioid misuse, injurious falls, and bleeding. Older adults are especially vulnerable to ADEs with nearly double the risk compared to younger populations [3]. Patients’ roles (including families in this protocol) are critical to medication safety in ambulatory settings [6-8]. An analysis of insulin-related emergency department visits identified patient self-management as the most common precipitant to insulin-related hypoglycemia and errors—patients incorrectly managed their food intake and insulin products at home [9]. Leading contributors to benzodiazepine adverse events were nonmedical use (56%) and self-harm (30%) [10].

Major gaps have been identified in patient and family engagement in medication safety in ambulatory settings [11-15], including cultural barriers that disrupt engagement between HCPs, patients, and families; lack of patients’ experience and skills in working with HCPs; and unclear expectations of patient roles in clinical encounters. Older adults often do not take advantage of existing systems for safe medication management practices at home, even for high-risk medications [16]. Patients often have an unvoiced agenda, especially related to concerns, side effects, and uncommunicated methods of managing their conditions [17]. Interventions to bridge these gaps in patient engagement include patient coaching prior to visits [18]; providing patients with instructional brochures, training videos, or prompts of questions to prepare for visits [19-21]; encouraging patients to bring medications and questions to visits [2]; and encouraging patients to ask questions [22-24]. One patient portal-based intervention focused on a dual approach of a previsit agenda-setting questionnaire followed by in-person coaching to prepare for encounters with HCPs [19].

This project uses the patient work system model [25]. The model, similar to those that study and improve the work performance of HCPs [26], focuses on the health-related work of patients and nonprofessionals. The concept of patient work systems broadens the scope for medication safety interventions to encompass patients’ home environments [25], including setting expectations and clarifying the roles of patients and families [27], using community resources [28], and incorporating patient perspectives in medication safety improvement [29].

Although the inventions reported so far address several gaps in patient and family engagement, integrated approaches are lacking to systematically target key elements of the joint patient-professional collaborative work to achieve productive interactions. A redesign that acts on multiple aspects of collaborative work is needed to help patients and HCPs set expectations for their partnership appropriate to each patient, along with tools to support collaborative work, and skills training appropriate for the time and resource constraints typical in primary care encounters. Specific aspects of collaborative work are not usually recognized, such as psychological safety in the mostly hierarchical patient-HCP relationship, severe limitation of time, lack of training and knowledge of teamwork concepts, and limited tools for self-efficacy.

### Objectives

The primary objective of this study is to assess the impact of a set of patient partnership tools that redesign primary care encounters to encourage and empower patients to make more effective use of those encounters to improve medication safety. Two notable features of the tools are (1) to encourage patients to tell, not just ask, primary care professionals (PCPs) about their medication use at home; and (2) to empower PCPs with tools to nudge patients to become active partners. The tools were developed to leverage the longitudinal relationship between patients and PCPs, instead of focusing on 1-time exposure to the tools. Comparison will be made between usual clinical processes as the control and redesigned clinic processes as the intervention.

## Methods

### Ethical Considerations

The human participant research protocol was approved by the University of Texas at Arlington Institutional Review Board under a reliance agreement for all study sites (protocol number and version UTA 2019-0439.25 approved on May 10, 2023; contact information: University of Texas at Arlington, Arlington, Texas, US, Angela Luna, IRB Specialist, Office of Regulatory Services, angela.luna@uta.edu). Documentation of patient consent was waived; verbal consent was sufficient and required for data collection (approved verbal consent scripts in Multimedia Appendix 1). The clinics determined that the interventions were part of their standard of care process once implemented. A data and safety monitoring board was appointed with one of the authors (RAY) as the lead to review any adverse events, protocol deviations, and issues with recruitment. The lead physician of each study site was included in the board. Quarterly reviews by the board will be conducted. The trial was registered with ClinicalTrials.gov (number NCT05880368 on May 26, 2023). The clinical leaders at each clinic will decide the types of primary care visits to be included in the interventions, such as annual wellness, disease management follow-up, or acute visits. The leaders will be asked to include all English- or Spanish-speaking patients who are at least 50 years old and have 5 or more medications listed in their electronic health records (EHRs). During each study period, requisite numbers of eligible patients will be recruited by a study coordinator to participate in data collection.

### Study Design

The protocol was developed according to the SPIRIT (Standard Protocol Items: Recommendations for Interventional Trials) statement (Multimedia Appendix 2) [30]. The study is a nonrandomized, cross-sectional stepped wedge cluster-controlled trial with 1 private family medicine clinic and 2 public safety-net primary care clinics each composing their own cluster (for a total of 3 clusters). There are 2 intervention sequences with 1 cluster per sequence and 1 control sequence with 1 cluster. We planned the study as a cluster trial because randomization of patients is not feasible when the clinical processes are changed for all patient visits once interventions are in place, not for individual visits. One control sequence is included in the clinic with no intervention planned.

### Study Settings

The 3 clinics were in urban settings in a Southwest metropolitan area of the United States. All clinics predominantly serve patients with low socioeconomic status. One clinic is private with 1 family medicine physician. The other 2 clinics are public and part of a safety-net health system with family medicine residency training programs with approximately 15 primary care providers. The clinical and administrative leaders agreed to participate and to the timelines for data collection and implementation.

### Interventions

Process redesign will be facilitated by two partnership tools: (1) a 1-page visit preparation guide given to relevant patients by clinic staff before seeing the provider, with the intention to improve communication and shared decision-making, and (2) a library of short educational videos that clinic staff encourage patients to watch on medication safety. The intervention design approach aimed to address multiple elements of collaborative work systems during primary care clinic visits to improve medication safety while minimizing the additional demands on busy PCPs and patients. The tools are expected to be used during typical patient wait times in the exam room and thus are expected to have minimal impact on throughputs. The following goals were considered during the design process: (1) to change collaborative work culture by clarifying patients’ roles in contributing to medication use preferences, information accuracy, and in being prepared to participate in shared decision-making; (2) to explicitly recognize fear and reluctance in patient-professional communication by using concepts from the psychological safety literature in teamwork; (3) to set an expectation and to support learning the basics of medication use, such as the refill process and knowledge about tools to reduce unintentional errors; and (4) to encourage problem-solving in community settings, such as contacting pharmacists, who are generally more accessible than primary care providers, with medication-related questions. Participatory design methodologies [31,32] were used in the design and formative evaluation of the tools with older adults and PCPs. Iterative testing was conducted with users, both patients and PCPs. A qualified medical writer wrote the scripts for the videos. Health literacy and patient education experts edited the final versions of the visit prep guide and the scripts for the videos.

The visit preparation guide (Multimedia Appendix 3) contains three sections: (“ask”) a list of question prompts for patients to consider about their medications, including one about deprescribing [33]; (“tell”) a list of prompts for patients to communicate their medication management views, practices, and concerns; and (“expect”) a set of behaviors to encourage collaborative work. The “ask” section had its origin in the approaches used in “Ask Me 3” [23] and EHR-based pre-encounter medication reconciliation [19], with a focus on barriers to self-efficacy in medication use. The “tell” section was designed to overcome communication barriers, such as fear of telling providers about nonadherence with medication regimens. The “expect” section is designed to provide means for providers to recognize and encourage patient collaborative actions, such as bringing medications to clinic visits [34].

The educational videos are less than 2 minutes each in length. The topics and learning objectives are based on interviews, focus groups, professional organization recommendations, and surveys with older adults and PCPs. The final set has 5 videos (length in min:sec): (1) working with your doctor (1:25); (2) taking medicine safely at home (1:33); (3) learning about your medicines (1:36); (4) working with your pharmacist (1:34); and (5) reading prescription labels (1:23). The videos are delivered in exam rooms on touchscreen tablets mounted to either the wall or portable stands.

There is no compensation to patients or clinicians for using the study tools. Participating clinics consider the interventions to be part of their standard of care once implemented, with no plans to compensate for any potential harm because of the trial.

### Strategies to Improve Adherence to Interventions

Five points (“moments”) of patient encounters during a typical office visit were identified to redesign clinical processes for enhanced patient partnership. The moments all present opportunities for PCPs to partner with patients to improve medication safety and were used to train PCPs on how to incorporate the interventions in the visit process. Two behavioral economics principles were used in developing sample scripts for PCPs: benefits appeal and psychological incentives [35].

1. Rooming, by medical assistants, sets the expectation for active partnership through benefits appeal and psychological incentives. Script examples include “This guide may help you be prepared. Check the items and give it to the doctor” and “I watched all these videos and I like them all. They are very short but will really help you.”
2. Greeting, by providers, recognizes and encourages preparation in the patient work system with psychological incentives. Script examples include “Did you bring your meds? Great. I like it when you are prepared!” and “I see you are trying to be prepared. Very good.”
3. Agenda setting, by providers, recognizes and encourages collaborative work through psychological incentives. Example scripts include “You used the prep guide. Thank you!” and “Thank you for telling me about your meds! I like it when we can work together better.”
4. Closing, by providers, recognizes and encourages patient learning and engagement with psychological incentives. Example scripts include “Did you watch the videos here? Do you want to learn more?” and “That was a good visit – you are a 5-star patient today.”
5. Discharge, by medical assistants or nurses, encourages learning through psychological incentives and group effects. Scripts examples include “It is great that you are trying to learn more about your meds” and “We can help you better by working together.”

At the discretion of the clinics, gifts of token values are given to patients by staff or providers as part of psychological incentives. Examples of gifts are water bottles, band-aid holders, pill boxes, and medication bags, with a bulk purchase value of about US $1 per item. Staff and providers are informed that although they are encouraged, they are free to decide whether to use the tools and to adapt the suggested partnership enhancement scripts in their clinic encounters.

### Outcomes

The primary outcome is self-efficacy in medication use in community settings. Self-efficacy will be measured by a validated tool: Medication Use and Self-Efficacy (MUSE) [36]. MUSE has eight items with a 5-point Likert scale, ranging from “1 – strongly disagree” to “5 – strongly agree”: (1) It is easy for me to take my medicine on time; (2) It is easy to remember to take all my medicines; (3) It is easy for me to set a schedule to take my medicines each day; (4) It is easy for me to take my medicines each day; (5) It is easy for me to ask my doctor questions about my medicine; (6) It is easy for me to understand my doctor’s instructions for my medicine; (7) It is easy for me to understand instructions on medicine bottles; and (8) It is easy for me to get all the information I need about my medicine. We replaced the word “pharmacist” with “doctor” given that our focus is on interventions in primary care settings. The MUSE score is the sum of the individual responses to the 8 items and ranges between 8 and 40, with 40 indicating “strongly agree” for all 8 items.

Secondary outcomes are a combination of patients’ and PCPs’ views on communication, collaborative work, and medication reviews. For patients, we will assess their views on collaborative work with seven items selected from five previously published instruments: (1) I know what each of my prescribed medications does (from Patient Activation Measure [37]); (2) I worry about drug interactions between the medications I take (from Medication-Related Problems [38]); (3) During the visit, I was asked to talk about any problems with my medicines or their effects (from Patient Assessment of Chronic Illness Care [39]); (4) During the visit, I was asked questions, either directly or on a survey, about my medicine habits (from Patient Assessment of Chronic Illness Care); (5) I understand what my doctor expects of me regarding my medicines (Psychological Safety Measure [40]); (6) If I make a mistake with my medicines, my doctor does not hold that against me (from Psychological Safety Measure); and (7) My doctor knows the vitamins and supplements I take (from Home Medication Experience Questionnaire [41]).

For providers, we will record the results of medication reviews in a medication review form and capture collaborative work during the visit. The medication review form was adapted from a toolkit published by the Agency for Health Care Research and Quality [42]. The medication review form measures regimen issues (contraindications, drug-drug interactions, and duplications); patient self-management issues (taking expired medications, taking incorrectly, failure to refill, and missed follow-up visits or lab testing); making changes without communicating with the primary care provider (making changes in medications, stopping a prescription, and stopping a supplement); and discrepancies in the medication record (taking medications not in the record, taking medications different from the record, medications active in the patient’s record but not taking, or incorrect dose information). For each issue identified, we asked the providers whether they believed it presented a risk to medication safety (no risk, minor risk, or major risk). The collaborative work activities captured are medication changes made in the visit (removal of expired medications, updating prescriptions, replacing prescriptions, prescribing new medications, regimen simplification, deprescribing, and changes in total number of medications); whether a caregiver is present, and if not, if a caregiver would be helpful; whether the patient brought their prescription and nonprescription medications to the visit; whether the patient knows why and how to take their medications; practices in engaging patients (agenda setting, creating or updating medication list with patients, and using teach-back techniques); and issues impeding the collaborative work (health literacy, language barriers, cognitive impairment, lack of a caregiver, not enough time, patient not knowing their medications).

All outcomes will be assessed immediately after the conclusion of a clinic visit. A research coordinator will survey consenting patients and give a paper form to the provider to collect data on primary and secondary outcomes. Implementation-related measures will be collected in terms of monthly use of the visit preparation guide and access logs of the video library. Protocol deviations will be reported and addressed per University of Texas at Arlington policies.

### Study Timeline

Cross-sectional measurements will be taken at 6 time periods of 6 weeks each and will include a transition period of no data collection for intervention clinics (Table 1). Sample sizes will vary by period and cluster. We powered the trial according to the stepped wedge design (clinics A and B only) so that the additional data from the control clinic would add to the power as a supplement. The control clinic is in the same health system as one of the intervention clinics and shares a similar patient population and staff. Using a stepped wedge design allows for the implementation of the intervention at different time points in each clinic, thus providing logistical advantages (like maximizing resources for implementation) and controlling for biases from trends in patient care [43].

**Table 1:** Design of the nonrandomized, cross-sectional stepped wedge cluster-controlled trial with the schedule of enrollment, interventions, and assessments.

Timepoint (6 weeks^a^)		Study period					
		*P*_1_	*P*_2_	*P*_3_	*P*_4_	*P*_5_	*P*_6_
**Study sites^b^**							
	Clinic A	CTRL^c^	N/A^d^	INT^e^	INT	INT	INT
	Clinic B	CTRL	CTRL	N/A	INT	INT	INT
	Clinic C	CTRL	CTRL	CTRL	CTRL	CTRL	CTRL
**Enrollment**							
	Eligibility screen	Clinics A, B, and C	Clinics B and C	Clinics A and C	Clinics A, B, and C	Clinics A, B, and C	Clinics A, B, and C
	Informed consent	Clinics A, B, and C	Clinics B and C	Clinics A and C	Clinics A, B, and C	Clinics A, B, and C	Clinics A, B, and C
**Interventions**							
	Visit Prep Guide	N/A	Clinic A	Clinics A and B	Clinics A and B	Clinics A and B	Clinics A and B
	Educational Videos	N/A	Clinic A	Clinics A and B	Clinics A and B	Clinics A and B	Clinics A and B
**Assessments**							
	Medication use self-efficacy	Clinics A, B, and C	Clinics B and C	Clinics A and C	Clinics A, B, and C	Clinics A, B, and C	Clinics A, B, and C
	Medication review	Clinics A, B, and C	Clinics B and C	Clinics A and C	Clinics A, B, and C	Clinics A, B, and C	Clinics A, B, and C
	Collaborative work	Clinics A, B, and C	Clinics B and C	Clinics A and C	Clinics A, B, and C	Clinics A, B, and C	Clinics A, B, and C

^a^Timepoint is in 6-week periods (*P*_1_-*P*_6_), total 36 weeks.

^b^Study sites (nonrandomized).

^c^CTRL: control.

^d^N/A: not applicable (transition period for implementation; no data collected).

^e^INT: intervention.

### Sample Size

The study is a superiority trial, on the hypothesis that interventions will improve patients’ self-efficacy in medication use. We powered the trial to detect a difference of 3.8 units on the MUSE scale from pre to postintervention, with a pooled SD of 4.7 (standardized effect size of 0.79) based on a validation study on MUSE [36] using a 2-tailed significance level of 0.05. Power analysis was computed using the R package (version 4.1.0; University of Washington) swCRTdesign [44] via a web-based tool [45]. The transition period was excluded from the sample size calculations. We used 0.07 for intracluster correction and 0.9 for cluster autocorrelation based on study design recommendations [46,47]. With power set at 80%, the requisite numbers of visits to collect data for each period are in Table 2, with a total sample size of 405.

**Table 2:** Sample size calculation. Sample size is the number of visits, not the number of patients, using a cross-sectional design.

	Period 1	Period 2	Period 3	Period 4	Period 5	Period 6
Clinic A	15	N/A^a^	35	20	15	15
Clinic B	30	50	N/A	45	30	30
Clinic C	20	20	20	20	20	20

^a^N/A: not applicable (transition period with no data collection efforts).

### Recruitment

During each 6-week data collection period, study coordinators will work with study clinics to select recruitment days and identify up to 4 eligible visits per provider on those days. Only 2 visits per provider per shift will be enrolled to avoid disrupting care. Providers will be recruited and consented, informed of identified visits on selected data collection days, and asked to obtain verbal consent from the patients in identified visits to be approached by the study coordinator. The coordinator will then recruit and consent the patient. Patient and clinician participants will be compensated US $10 and US $25, respectively, for each data collection encounter.

### Assignment of Interventions and Blinding

Because of the nature of the assessed interventions, we consider blinding not possible for patients or PCPs. Research coordinators who collect data are not blinded to the interventions as they may see interventions, but they are instructed to conduct data collection in consistent procedures regardless of intervention status.

### Data Management and Confidentiality

All primary and secondary data collected will be entered into Research Electronic Data Capture (REDCap; Vanderbilt). A quarterly data review will be carried out to assess potential issues with data collection, including missing data and duplicate records. No personal identifying data will be collected.

### Statistical Methods for Primary and Secondary Outcomes

Hierarchical models will be used to assess the impact of the interventions on primary and secondary outcomes. Hierarchical models, including linear mixed models, have been described and recommended for use in testing for intervention effects in cross-sectional stepped wedge designs [48,49]. The effect of the intervention is subject to confounding with time due to the staggered entry in stepped wedge designs, and thus the hierarchical modeling structure captures time trends and accounts for the effects of clustering within clinics. Should there be ceiling or floor effects with the total MUSE scores, responses to individual items will be explored. No interim analysis will be conducted. Social determinants of health will be evaluated, including sex, age, race, ethnicity, preferred language, educational level attained, self-reported health literacy, insurance status, and study clinics (private vs public). Associations of these variables with primary outcomes will be assessed, as we anticipate social determinants of health may affect the impact of the interventions. Selected social determinants of health will be assessed through subgroup analyses and model adjustment.

Descriptive statistics, such as medians, IQRs, and proportions, will be tabulated, overall and within clinics, to understand the distribution of the study population. Potential confounding variables were specified prior to data collection and include patient demographics, social determinants of health, time, and clinician. Confounding variables will be evaluated and controlled for in the analysis primarily through model adjustment or stratification. Because the intervention is confounded by time, the model will account for time period as a covariate in the statistical model, following recommendations on mixed models for stepped wedge trials by Li et al [48]. In addition, we included an extra clinic as a control group to assess changes over time in the absence of the intervention.

The study protocol is publicly registered and fully available. Participant level-data will not be made available. Statistical code will be made available upon request.

### Dissemination Plan

A writing group will lead publication efforts in peer-reviewed journals and professional conferences. Reports will follow the CONSORT (Consolidated Standards of Reporting Trials) statement and its extensions (for both cluster randomized trials and nonpharmacological interventions) and follow journal authorship guidelines. We will also report implementation-related findings such as uptake patterns of tools over time and qualitative feedback from clinicians and patients at the study sites.

## Results

The study was funded in September 2019. The trial started on April 10, 2023, and continued until December 15, 2023. The 3 study sites were in urban settings in a Southwest metropolitan area of the United States. All clinics predominantly serve patients with low socioeconomic status. One clinic is private with 1 family medicine physician. The other 2 clinics are public and part of a safety-net health system with family medicine residency training programs with approximately 15 primary care providers. As of February 15, 2024, we enrolled 405 patients. Data analysis is currently underway and the first results are expected to be submitted for publication in 2024.

## Discussion

The trial aims to assess the impact of a set of patient partnership tools that redesign primary care encounters on patient self-efficacy in medication use in community settings. Secondarily, the trial will provide information on the systematic approach to patient partnership enhancement in collaborative work during primary care visits, from both the patients’ and providers’ perspectives. We expect the tools and associated process changes in primary care clinics to improve several components of the collaborative work system in terms of self-efficacy, tools, and skills. The intervention is unique compared with prior approaches, such as generic videos on open communication between patients and HCPs or EHR-based tools to help agenda setting [19,22]. If adoption proves successful and MUSE scores rise, the trial will support the innovative approach to improve medication safety in ambulatory settings by improving the value of primary care through collaborative work and partnership. The trial will be carried out in clinics serving vulnerable populations with poor indicators for social determinants of health. The combination of a private family medicine clinic and a primary care clinic in a public safety-net hospital system will provide evidence about the applicability of the approach in a wide range of clinical settings. In addition to the visit prep guide and videos, the trial will also generate other tools for implementation, such as training for PCPs to engage the patient work system and thus improve self-efficacy and ultimately medication safety.

Both the intervention and the assessment approaches are based on collaborative work concepts not previously used to increase patient engagement. Psychological safety in the hierarchical relationship between patients and providers and the clarification of roles and responsibilities have been absent in prior interventions. Although prescribing decisions are important aspects of medication safety, the intervention targets barriers to successful self-management in the patient work system, including patients’ home environments.

The trial design has several strengths. The assessment collects data to illuminate both the patient’s and PCP’s perspectives immediately after an office visit. The data include both patient-centered safety outcomes such as self-efficacy and clinical outcomes such as risks posed by issues identified during medication review. The data also include process measures on collaborative work like psychological safety. The studied patient population is on 5 or more chronic medications and thus is a high-risk group.

The trial has several limitations. Data on ADEs are not collected. Future studies are needed to assess the longer-term impact. The trial is limited in time and thus longitudinal data are not collected on the potential long-term impact of the partnership tools (ie, the impact of repeated exposure over years). The modality of tools (paper visit preparation guides and exam room videos on tablets) is limited and does not leverage consumer and EHR technology in communication and in learning. The number of study clinics is small. However, with stepped wedge design, we expect to control some of the confounding variables, such as seasonality.

## Appendix

Multimedia Appendix 1 Verbal consent scripts.

Multimedia Appendix 2 SPIRIT (Standard Protocol Items: Recommendations for Interventional Trials) checklist.

Multimedia Appendix 3 Patient visit guide (English).

## Abbreviations

ADE: adverse drug event
CONSORT: Consolidated Standards of Reporting Trials
EHR: electronic health record
HCP: health care professional
MUSE: Medication Use and Self-Efficacy
PCP: primary care professional
REDCap: Research Electronic Data Capture
SPIRIT: Standard Protocol Items: Recommendations for Interventional Trials

## Data Availability

The data sets generated and analyzed during this study are not publicly available to protect patient confidentiality but may be available from the corresponding author upon reasonable request. The assessment instruments and intervention tools are available for noncommercial use.
