# Supplementary material for: Patient Partnership Tools to Support Medication Safety in Community-Dwelling Older Adults: Protocol for a Nonrandomized Stepped Wedge Clinical Trial
Source: JMIR Res Protoc. 2024 Apr 29;13:e57878. doi: 10.2196/57878 (PMC11091807; doi:10.2196/57878)
Supplement: Multimedia Appendix 1 [file resprot_v13i1e57878_app1.pdf]

The University of Texas at Arlington (UTA)

Verbal Consent Script for Post Visit Survey

I am asking you to participate in a UT Arlington research study titled "Partnership in Resilience for Medication Safety." We are asking you to tell us about your visit experience today in a survey. This will take approximately 5 minutes. As compensation for your participation, you will be entitled to receive a $25 Amazon gift card.

The survey is not expected to pose any risks beyond those that you would experience in your everyday life or during routine medical visits. The only potential risk to you would be a breach of confidentiality. However, the study investigators will take all precautions necessary to protect your confidentiality.

Your participation and answers will not affect your status at this clinic. Your responses will not be shared with the clinic.

The decision to participate is entirely yours. If you feel uncomfortable answering some of the questions in the survey, you may skip them. You can also quit the survey at any time without any consequences.

Do you have any questions before proceeding to answering the survey?

You are indicating your voluntary agreement to participate in the PROMIS research project by beginning this survey.

The University of Texas at Arlington (UTA)

Verbal Consent Script for Post Visit Survey

I am asking you to participate in a UT Arlington research study titled "Partnership in Resilience for Medication Safety." We are asking you to tell us about your visit experience today in a survey. This will take approximately 10 minutes. As compensation for your participation, you will be entitled to receive a $10 Walmart gift card.

The survey is not expected to pose any risks beyond those that you would experience in your everyday life or during routine medical visits. The only potential risk to you would be a breach of confidentiality. However, the study investigators will take all precautions necessary to protect your confidentiality.

Your participation and answers will not affect the care you receive at this clinic. Your responses will not be shared with your provider or the clinic.

The decision to participate is entirely yours. If you feel uncomfortable answering some of the questions in the survey, you may skip them. You can also quit the survey at any time without any consequences.

Do you have any questions before proceeding to answering the survey?

You are indicating your voluntary agreement to participate in the PROMIS research project by beginning this survey.
