# Supplementary material for: Patient Partnership Tools to Support Medication Safety in Community-Dwelling Older Adults: Protocol for a Nonrandomized Stepped Wedge Clinical Trial
Source: JMIR Res Protoc. 2024 Apr 29;13:e57878. doi: 10.2196/57878 (PMC11091807; doi:10.2196/57878)
Supplement: Multimedia Appendix 2 [file resprot_v13i1e57878_app2.docx]

| Standard Protocol Items: Recommendations for Interventional Trials (SPIRIT) Checklist [Source: Chan A, Tetzlaff JM, Gøtzsche PC, Altman DG, Mann H, Berlin JA, et al. SPIRIT 2013 explanation and elaboration: guidance for protocols of clinical trials. BMJ. 2013;346:e7586. https:// doi. org/ 10. 1136/ bmj. e7586] | | | | |
| --- | --- | --- | --- | --- |
| *Item* | *#* | *Description* | *Status* | |
| Title | 1 | Descriptive title identifying the study design, population, interventions, and, if applicable, trial acronym | Okay – in compliance with JMIR’s guide on titles | |
| Trial registration | 2a | Trial identifier and registry name. If not yet registered, name of intended registry | ClinicalTrials.gov registration information in Methods section | |
| Trial registration: data set | 2b | All items from the World Health Organization Trial Registration Data Set | Trial was registered with the required elements. | |
| Protocol version | 3 | Date and version identifier | Protocol version in Method section | |
| Funding | [4](https://www.goodreports.org/reporting-checklists/spirit/info/#4) | Sources and types of financial, material, and other support | Funding information in acknowledgment section | |
| Roles and responsibilities: contributorship | 5a | Names, affiliations, and roles of protocol contributors | Authors and contributions listed in author list and in contribution section | |
| Roles and responsibilities: sponsor contact information | 5b | Name and contact information for the trial sponsor | Contact information provided in Method section | |
| Roles and responsibilities: sponsor and funder | 5c | Role of study sponsor and funders, if any, in study design; collection, management, analysis, and interpretation of data; writing of the report; and the decision to submit the report for publication, including whether they will have ultimate authority over any of these activities | Acknowledgment section declares the role of sponsors. | |
| Roles and responsibilities: committees | 5d | Composition, roles, and responsibilities of the coordinating center, steering committee, endpoint adjudication committee, data management team, and other individuals or groups overseeing the trial, if applicable (see Item 21a for data monitoring committee) | Methods section under “Ethical Considerations” | |
| **Introduction** |  |  |  |  |
| Background and rationale | 6a | Description of research question and justification for undertaking the trial, including summary of relevant studies (published and unpublished) examining benefits and harms for each intervention | Subsection in Introduction | |
| Background and rationale: choice of comparators | 6b | Explanation for choice of comparators | Introduction under “Objectives” | |
| Objectives | 7 | Specific objectives or hypotheses | Subsection in Introduction | |
| Trial design | 8 | Description of trial design including type of trial (eg, parallel group, crossover, factorial, single group), allocation ratio, and framework (eg, superiority, equivalence, non-inferiority, exploratory) | In “Study design” in Methods | |
| **Methods: Participants, interventions, and outcomes** | | | | |
| Study setting | 9 | Description of study settings (eg, community clinic, academic hospital) and list of countries where data will be collected. Reference to where list of study sites can be obtained | Subsection under methods section | |
| Eligibility criteria | 10 | Inclusion and exclusion criteria for participants. If applicable, eligibility criteria for study centers and individuals who will perform the interventions (eg, surgeons, psychotherapists) | Methods section under “eligibility criteria and informed consent” | |
| Interventions: description | 11a | Interventions for each group with sufficient detail to allow replication, including how and when they will be administered | Methods section | |
| Interventions: modifications | 11b | Criteria for discontinuing or modifying allocated interventions for a given trial participant (eg, drug dose change in response to harms, participant request, or improving / worsening disease) | Not applicable (no modification) | |
| Interventions: adherence | 11c | Strategies to improve adherence to intervention protocols, and any procedures for monitoring adherence (eg, drug tablet return; laboratory tests) | Subsection under methods section | |
| Interventions: concomitant care | 11d | Relevant concomitant care and interventions that are permitted or prohibited during the trial | In “Interventions” subsection under methods section | |
| Outcomes | 12 | Primary, secondary, and other outcomes, including the specific measurement variable (eg, systolic blood pressure), analysis metric (eg, change from baseline, final value, time to event), method of aggregation (eg, median, proportion), and time point for each outcome. Explanation of the clinical relevance of chosen efficacy and harm outcomes is strongly recommended | Subsection under methods section | |
| Participant timeline | [13](https://www.goodreports.org/reporting-checklists/spirit/info/#13) | Time schedule of enrolment, interventions (including any run-ins and washouts), assessments, and visits for participants. A schematic diagram is highly recommended (see Figure) | Subsection under methods section | |
| Sample size | [14](https://www.goodreports.org/reporting-checklists/spirit/info/#14) | Estimated number of participants needed to achieve study objectives and how it was determined, including clinical and statistical assumptions supporting any sample size calculations | Subsection under methods section | |
| Recruitment | [15](https://www.goodreports.org/reporting-checklists/spirit/info/#15) | Strategies for achieving adequate participant enrolment to reach target sample size | Subsection under methods section | |
| **Methods: Assignment of interventions (for controlled trials)** | | | | |
| Allocation: sequence generation | [16a](https://www.goodreports.org/reporting-checklists/spirit/info/#16a) | Method of generating the allocation sequence (eg, computer-generated random numbers), and list of any factors for stratification. To reduce predictability of a random sequence, details of any planned restriction (eg, blocking) should be provided in a separate document that is unavailable to those who enroll participants or assign interventions | “Assignment of interventions and blinding” under methods section | |
| Allocation concealment mechanism | [16b](https://www.goodreports.org/reporting-checklists/spirit/info/#16b) | Mechanism of implementing the allocation sequence (eg, central telephone; sequentially numbered, opaque, sealed envelopes), describing any steps to conceal the sequence until interventions are assigned | “Assignment of interventions and blinding” under methods section | |
| Allocation: implementation | [16c](https://www.goodreports.org/reporting-checklists/spirit/info/#16c) | Who will generate the allocation sequence, who will enroll participants, and who will assign participants to interventions | “Assignment of interventions and blinding” under methods section | |
| Blinding (masking) | [17a](https://www.goodreports.org/reporting-checklists/spirit/info/#17a) | Who will be blinded after assignment to interventions (eg, trial participants, care providers, outcome assessors, data analysts), and how | “Assignment of interventions and blinding” under methods section | |
| Blinding (masking): emergency unblinding | [17b](https://www.goodreports.org/reporting-checklists/spirit/info/#17b) | If blinded, circumstances under which unblinding is permissible, and procedure for revealing a participant’s allocated intervention during the trial | “Assignment of interventions and blinding” under methods section | |
| **Methods: Data collection, management, and analysis** | | | | |
| Data collection plan | [18a](https://www.goodreports.org/reporting-checklists/spirit/info/#18a) | Plans for assessment and collection of outcome, baseline, and other trial data, including any related processes to promote data quality (eg, duplicate measurements, training of assessors) and a description of study instruments (eg, questionnaires, laboratory tests) along with their reliability and validity, if known. Reference to where data collection forms can be found, if not in the protocol | “Outcomes” under methods section | |
| Data collection plan: retention | [18b](https://www.goodreports.org/reporting-checklists/spirit/info/#18b) | Plans to promote participant retention and complete follow-up, including list of any outcome data to be collected for participants who discontinue or deviate from intervention protocols | Not applicable (One time data collection) | |
| Data management | [19](https://www.goodreports.org/reporting-checklists/spirit/info/#19) | Plans for data entry, coding, security, and storage, including any related processes to promote data quality (eg, double data entry; range checks for data values). Reference to where details of data management procedures can be found, if not in the protocol | “Data management and confidentiality” under methods section | |
| Statistics: outcomes | [20a](https://www.goodreports.org/reporting-checklists/spirit/info/#20a) | Statistical methods for analyzing primary and secondary outcomes. Reference to where other details of the statistical analysis plan can be found, if not in the protocol | In “Statistical methods for primary and secondary outcomes” in methods section | |
| Statistics: additional analyses | [20b](https://www.goodreports.org/reporting-checklists/spirit/info/#20b) | Methods for any additional analyses (eg, subgroup and adjusted analyses) | In “Statistical methods for primary and secondary outcomes” in methods section | |
| Statistics: analysis population and missing data | [20c](https://www.goodreports.org/reporting-checklists/spirit/info/#20c) | Definition of analysis population relating to protocol non-adherence (eg, as randomized analysis), and any statistical methods to handle missing data (eg, multiple imputation) | In “Statistical methods for primary and secondary outcomes” in methods section | |
| **Methods: Monitoring** | | | | |
| Data monitoring: formal committee | [21a](https://www.goodreports.org/reporting-checklists/spirit/info/#21a) | Composition of data monitoring committee (DMC); summary of its role and reporting structure; statement of whether it is independent from the sponsor and competing interests; and reference to where further details about its charter can be found, if not in the protocol. Alternatively, an explanation of why a DMC is not needed | In “Ethical Considerations” in Methods section | |
| Data monitoring: interim analysis | [21b](https://www.goodreports.org/reporting-checklists/spirit/info/#21b) | Description of any interim analyses and stopping guidelines, including who will have access to these interim results and make the final decision to terminate the trial | In “Statistical Methods for Primary and Secondary Outcomes” in Methods section (“No interim analysis”) | |
| Harms | [22](https://www.goodreports.org/reporting-checklists/spirit/info/#22) | Plans for collecting, assessing, reporting, and managing solicited and spontaneously reported adverse events and other unintended effects of trial interventions or trial conduct | In “Ethical Considerations” in Methods section | |
| Auditing | [23](https://www.goodreports.org/reporting-checklists/spirit/info/#23) | Frequency and procedures for auditing trial conduct, if any, and whether the process will be independent from investigators and the sponsor | In “Ethical Considerations” in Methods section | |
| **Ethics and dissemination** | | | | |
| Research ethics approval | [24](https://www.goodreports.org/reporting-checklists/spirit/info/#24) | Plans for seeking research ethics committee / institutional review board (REC / IRB) approval | In “Ethical Considerations” in Methods section | |
| Protocol amendments | [25](https://www.goodreports.org/reporting-checklists/spirit/info/#25) | Plans for communicating important protocol modifications (eg, changes to eligibility criteria, outcomes, analyses) to relevant parties (eg, investigators, REC / IRBs, trial participants, trial registries, journals, regulators) | In “Ethical Considerations” in Methods section | |
| Consent or assent | [26a](https://www.goodreports.org/reporting-checklists/spirit/info/#26a) | Who will obtain informed consent or assent from potential trial participants or authorized surrogates, and how (see Item 32) | Methods section under “eligibility criteria and informed consent” | |
| Consent or assent: ancillary studies | [26b](https://www.goodreports.org/reporting-checklists/spirit/info/#26b) | Additional consent provisions for collection and use of participant data and biological specimens in ancillary studies, if applicable | Not applicable | |
| Confidentiality | [27](https://www.goodreports.org/reporting-checklists/spirit/info/#27) | How personal information about potential and enrolled participants will be collected, shared, and maintained in order to protect confidentiality before, during, and after the trial | “Data management and confidentiality” under methods section | |
| Declaration of interests | [28](https://www.goodreports.org/reporting-checklists/spirit/info/#28) | Financial and other competing interests for principal investigators for the overall trial and each study site | “Competing interests” as part of end matter | |
| Data access | [29](https://www.goodreports.org/reporting-checklists/spirit/info/#29) | Statement of who will have access to the final trial dataset, and disclosure of contractual agreements that limit such access for investigators | “Availability of data and materials” as part of end matter | |
| Ancillary and post trial care | [30](https://www.goodreports.org/reporting-checklists/spirit/info/#30) | Provisions, if any, for ancillary and post-trial care, and for compensation to those who suffer harm from trial participation | In “Interventions” subsection under methods section | |
| Dissemination policy: trial results | [31a](https://www.goodreports.org/reporting-checklists/spirit/info/#31a) | Plans for investigators and sponsor to communicate trial results to participants, healthcare professionals, the public, and other relevant groups (eg, via publication, reporting in results databases, or other data sharing arrangements), including any publication restrictions | Subsection under methods section | |
| Dissemination policy: authorship | [31b](https://www.goodreports.org/reporting-checklists/spirit/info/#31b) | Authorship eligibility guidelines and any intended use of professional writers | “Author contributions” as part of the end matter | |
| Dissemination policy: reproducible research | [31c](https://www.goodreports.org/reporting-checklists/spirit/info/#31c) | Plans, if any, for granting public access to the full protocol, participant-level dataset, and statistical code | In “Statistical methods for primary and secondary outcomes” in methods section | |
| **Appendices** | | | | |
| Informed consent materials | [32](https://www.goodreports.org/reporting-checklists/spirit/info/#32) | Model consent form and other related documentation given to participants and authorized surrogates | Approved verbal consent scripts are in Appendix | |
| Biological specimens | [33](https://www.goodreports.org/reporting-checklists/spirit/info/#33) | Plans for collection, laboratory evaluation, and storage of biological specimens for genetic or molecular analysis in the current trial and for future use in ancillary studies, if applicable | Not applicable | |
