# Supplementary material for: Patient Partnership Tools to Support Medication Safety in Community-Dwelling Older Adults: Protocol for a Nonrandomized Stepped Wedge Clinical Trial
Source: JMIR Res Protoc. 2024 Apr 29;13:e57878. doi: 10.2196/57878 (PMC11091807; doi:10.2196/57878)
Supplement: Multimedia Appendix 3 [file resprot_v13i1e57878_app3.pdf]

Staff use: Date ________ Age: <50 50-64 ≥65 # of Meds: 0 1-4 5-10 >10
Gender: F___ M___ Other___ Race/Ethnicity_______Study ID if assigned: _____________

Visit Prep Guide - Medicines

Your doctor would like for you to fill out this guide –

Working together to get most of this visit.

▪ Ask your doctor - Check 1-3 questions you want to ask today:

- ☐ What happens if I stop taking this medicine? _____________
- ☐ What can I do to lower the side effects of this medicine? _________
- ☐ Can I take fewer medicines than I am taking?
- ▪ **For conditions like diabetes, heart conditions, high blood pressure:**
- ☐ How can I stop my blood sugar, heart rate, or blood pressure from getting too low?
- ☐ Why do I need several medicines for my condition?
- ☐ What should I eat, and what should I not eat for my condition?
- ☐ How do I learn more about my condition?
- ☐ Other questions: ____

▪ Tell your doctor – Check 1-3 things you want to talk about medicines:

- ☐ I have new medicines from other doctors (offices, hospitals or emergency rooms)
- ☐ I stopped or skipped these medicines _______________________________________
  Due to ☐ Cost ☐ Side effects ☐ Other reasons ________________
- ☐ I have trouble getting this medicine _____________
- ☐ I often forget to take my medicines
- ☐ I get my medicines from several places
- ☐ I have concerns with my medicines (examples: cost, hard to read, not helping much)
- ☐ I have a good routine (examples: pillboxes, helper setting them up, reminder alarms)
- ☐ I learned new things recently (examples: reading medicine labels, using pillboxes, calling a pharmacist)

▪ 5 Star Actions! We love it when patients do these. Which are you doing?

- ☐ I bring my medicine containers to doctor's visits
- ☐ I bring the list of my medicines to doctor's visits
- ☐ I write down questions to ask my doctor
- ☐ I have things to tell my doctor about my medicines
